# Development of a process to disclose amyloid imaging results to cognitively normal older adult research participants

**DOI:** 10.1186/s13195-015-0112-7

**Published:** 2015-05-12

**Authors:** Kristin Harkins, Pamela Sankar, Reisa Sperling, Joshua D Grill, Robert C Green, Keith A Johnson, Megan Healy, Jason Karlawish

**Affiliations:** Department of Medicine, University of Pennsylvania Perelman School of Medicine, Philadelphia, PA USA; Department of Medical Ethics and Health Policy, University of Pennsylvania Perelman School of Medicine, Philadelphia, PA USA; Department of Neurology, Harvard Medical School, Center for Alzheimer Research and Treatment, Brigham & Women’s Hospital Massachusetts ADRC, Massachusetts General Hospital, Boston, MA USA; Mary S. Easton Center for Alzheimer’s Disease Research, University of California Los Angeles, Los Angeles, CA USA; Department of Medicine, Division of Genetics, Brigham & Women’s Hospital and Harvard Medical School, Boston, MA USA; Departments of Neurology and Radiology, Harvard Medical School, Center for Alzheimer Research and Treatment, Brigham & Women’s Hospital Massachusetts ADRC, Massachusetts General Hospital, Boston, MA USA; Departments of Medicine, Medical Ethics & Health Policy, University of Pennsylvania Perelman School of Medicine, Penn Neurodegenerative Disease Ethics & Policy Program, University of Pennsylvania, 3615 Chestnut St, Philadelphia, PA 19104 USA

## Abstract

**Introduction:**

The objective of this study was to develop a process to maximize the safety and effectiveness of disclosing Positron Emission Tomography (PET) amyloid imaging results to cognitively normal older adults participating in Alzheimer’s disease secondary prevention studies such as the Anti-Amyloid Treatment in Asymptomatic Alzheimer’s Disease (A4) Study.

**Methods:**

Using a modified Delphi Method to develop consensus on best practices, we gathered and analyzed data over three rounds from experts in two relevant fields: informed consent for genetic testing or human amyloid imaging.

**Results:**

Experts reached consensus on (1) text for a brochure that describes amyloid imaging to a person who is considering whether to undergo such imaging in the context of a clinical trial, and (2) a process for amyloid PET result disclosure within such trials. Recommendations included: During consent, potential participants should complete an educational session, where they receive verbal and written information covering what is known and unknown about amyloid imaging, including possible results and their meaning, implications of results for risk of future cognitive decline, and information about Alzheimer’s and risk factors. Participants should be screened for anxiety and depression to determine suitability to receive amyloid imaging information. The person conducting the sessions should check comprehension and be skilled in communication and recognizing distress. Imaging should occur on a separate day from consent, and disclosure on a separate day from imaging. Disclosure should occur in person, with time for questions. At disclosure, investigators should assess mood and willingness to receive results, and provide a written results report. Telephone follow-up within a few days should assess the impact of disclosure, and periodic scheduled assessments of depression and anxiety, with additional monitoring and follow-up for participants showing distress, should be performed.

**Conclusions:**

We developed a document for use with potential study participants to describe the process of amyloid imaging and the implications of amyloid imaging results; and a disclosure process with attention to ongoing monitoring of both mood and safety to receive this information. This document and process will be used in the A4 Study and can be adapted for other research settings.

## Introduction

Progress in Alzheimer’s disease (AD) biomarker discovery has begun to transform how researchers and clinicians define the disease. Researchers have proposed that AD biomarkers are present prior to cognitive impairment and associate with subsequent cognitive and functional decline, supporting the concept of a ‘preclinical’ stage of AD [1]. Among the candidate biomarkers to identify this stage of AD, detection of fibrillar forms of amyloid-beta using positron emission tomography (PET) imaging has attracted considerable attention. Amyloid build up may be among the first pathological changes in AD and amyloid PET imaging may allow the identification of individuals at risk for progression to AD dementia and in whom targeted interventions to prevent that disability can be tested [2].

Among the studies needed to validate this stage of AD are randomized and controlled clinical trials that test whether intervening in cognitively normal persons who have a biomarker delays the onset of, or alters the rate of, cognitive decline. Such secondary prevention trials will enroll persons meeting preclinical AD criteria to test the efficacy of experimental compounds. These trials serve an important public health goal, as they will contribute to the National Alzheimer’s Project Act (NAPA) goal to prevent AD by 2025 [3]. One example of a secondary prevention trial is the Alzheimer’s Disease Cooperative Study (ADCS) Anti-Amyloid Treatment in Asymptomatic Alzheimer’s Study, or A4 Study [4].

The A4 Study will randomize 1,000 individuals with elevated brain amyloid as seen on PET scan to receive either a monoclonal antibody against the amyloid beta protein or placebo. Compared to a secondary prevention trial that includes a biomarker negative cohort blinded and randomized to placebo, this approach reduces the number of participants needed to enroll and avoids subjecting individuals to study procedures that are unlikely to benefit them [5]. It also allows researchers to study how telling a person their biomarker status impacts their cognition, cognitive symptoms, and well-being in the context of participating in a clinical trial that tests an intervention for biomarker positive persons.

As valuable as this approach may be, disclosing AD biomarker results to cognitively normal older adults does raise important clinical and ethical challenges. PET amyloid imaging is Food and Drug Administration (FDA) approved for the diagnostic work up of patients with progressive cognitive impairment, and appropriate use criteria recommend it only for persons with mild cognitive impairment (MCI) or dementia with an unclear or atypical cause. It is not recommended for cognitively normal individuals in the clinical setting [6]. Disclosure of AD biomarker results in a research context has been discouraged due to lack of clinical utility and treatment options and the prognostic uncertainty in this population. Studies suggest that up to 30% of cognitively normal older adults are amyloid positive, and at elevated risk of developing AD symptoms, but their individual risk of developing AD symptoms is not known [7]. A survey of Alzheimer’s Disease Neuroimaging Initiative (ADNI) researchers showed that both expert clinicians who supported and did not support disclosing amyloid imaging results recognized a need for a process to disclose safely and effectively and to study the impact of disclosure [8].

The goal of this study was to develop such a process and to test the disclosure language in a sample of volunteers. The project in its entirety was a component of the protocol development process for the A4 Study. We sought to answer the following questions: What information should an investigator disclose to AD prevention trial participants pre- and post- amyloid imaging? What are best practices for pre- and post- amyloid imaging discussions? What should be measured to assess the impact of learning this information in a clinical trial?

We utilized a modified Delphi method to develop expert consensus on best practices for amyloid results disclosure in AD prevention trials [9]. Expert consensus is recognized as a viable method of providing a basis for decision-making in situations where evidence from other sources, such as randomized trials, is sparse or non-existent [10].

## Methods

### Expert identification

The modified Delphi method we employed required an expert panel that included researchers from each of two relevant fields: informed consent for genetic testing and AD biomarkers with a focus on amyloid imaging in humans. To assure that individuals recruited for the expert panel were chosen using objective criteria, a version of the knowledge resource nomination method based on a strategic literature search was employed [9]. The study required that experts be willing to participate in a three-stage Delphi process.

The strategic literature search sought experts in informed consent for genetic testing and AD biomarkers with a focus on amyloid imaging in humans. The need for experts in amyloid imaging is self-evident. We sought experts in informed consent for genetic testing because amyloid imaging in clinically normal individuals is analogous to a genetic test in so far as it represents a biological measure that is associated with the later risk of a disease.

We defined an expert as an individual whose PubMed citations showed a consistent pattern of publication in either informed consent or amyloid imaging. We defined a consistent pattern as at least three years of publications with attention to lead or senior author status and the quality of the publications based on journal type and citations of the publication. Our target was to enroll 10 to 12 experts equally distributed between the two fields, following suggestions in the literature that an expert panel should include the minimally sufficient number of respondents and that a panel of 10 to 15 experts is adequate for relatively homogenous groups [11].

To identify experts in the field of amyloid imaging and AD biomarkers, we searched Medline for recent and relevant publications, performing searches for keywords: ‘amyloid imaging’, ‘Alzheimer Disease (subheading ‘ri (Radionuclide Imaging)’)’, and combinations of keywords ‘Alzheimer Disease’ AND ‘amyloid imaging’. All searches were limited to publications written in English from the year 2000 to 2012.

For the search to find experts on informed consent and genetic testing, we conducted Medline searches for recent and relevant publications, using keywords ‘Informed Consent’, ‘Genetic Testing’, and ‘Ethics’ and combinations of these keywords. All searches were limited to publications written in English from the year 2000 to 2012.

We reviewed the search results and identified potential expert participants based on the criteria described above. We produced an initial list of 20 individuals with consistent publication records in amyloid imaging and 20 individuals with consistent publication records in informed consent. We then randomly arranged the list of experts and serially recruited them in batches until we achieved our desired numbers.

We solicited expert participation via email that described the three rounds of Delphi review and then sent letters to those who did not respond or for whom email addresses were not available. Follow up letters were sent two weeks after the initial solicitation. After three attempted contacts, it was assumed that an individual did not wish to participate.

### Delphi round 1

All experts who agreed to participate were sent a PowerPoint slide set describing the basics of amyloid imaging and its role in trials such as the A4 Study. Experts then participated in one-on-one semi-structured telephone interviews with a trained research assistant (copies of the slide set and interview script are available upon request). The research assistant confirmed at the beginning of the interview that the expert had reviewed the PowerPoint slides. If not, the interview was rescheduled. The interviewer answered any questions about the slides. Experts were then asked questions to elicit their ideas about the process and topics to be covered during three different phases of the A4 study: before consent, after consent but prior to amyloid imaging, and after amyloid imaging. The goal of this step was to identify potential disclosure topics and an outline of the disclosure process. We transcribed interviews and reviewed responses to identify and remove duplicate responses, and standardize language. We organized responses into the following domains: topics, methods/steps, assessments, and materials for education and assessment. Each domain was again separated into three phases: before consent, after consent and after imaging.

### Delphi round 2

We sent the revised response list to experts via an online survey. For each item, experts used a three point scale (should be included, unsure, should not be included) to rate the necessity/appropriateness of including it. We also provided space for experts to comment on the reasons for their ratings.

We compiled the responses and categorized items into three levels of support: consensus to include (≥80% support), mixed support (79% to 50% support) and not supported (<50% support). Mixed support items were construed as having been supported if the majority of remaining votes were ‘unsure’ rather than ‘do not include’.

We used the list of items with consensus support to draft text for an Amyloid Imaging Disclosure Process Instructional Manual (hereafter called ‘the instructional manual’) and to create an Amyloid Imaging Disclosure Process brochure (hereafter called ‘the brochure’). The instructional manual is intended for investigators and clinicians, and describes each topic and creates a template for the process of amyloid result disclosure in the context of the A4 Study. The brochure describes amyloid imaging and is intended for education of a person who is considering whether to undergo it in the context of a clinical trial. We subsequently revised and refined the text in both drafts in collaboration with A4 investigators.

### Delphi round 3

We sent the draft brochure text to experts via an online survey. Experts rated each section (and the overall document) for clarity on a 1 to 5 scale (‘not at all clear’ to ‘extremely clear’). We provided space for experts to comment on the reasons for their ratings or provide suggestions for additional topics, changes and deletions. We used expert comments to revise the brochure, with particular attention to any sections with mean clarity ratings less than 4.

### Examine readability with cognitively normal older adults

Based on the results of Round 3, we tested a template version of the brochure that was not specific to the A4 study with a group of five cognitively normal older adults. Participants were a convenience sample recruited from the University of Pennsylvania Alzheimer’s Disease Center’s normal control cohort, who had given permission to be contacted for studies such as this one. We selected these persons because they would be the kind of person recruited for an AD secondary prevention trial. We asked participants to review the brochure prior to a face-to-face meeting with a trained research assistant. The research assistant assessed understanding using standardized measures developed in the decisional capacity literature that asked persons to ‘say back’ the meaning of a section, and reviewed sections with poor understanding for suggestions on improving clarity.

### Human subjects’ protections

The Delphi study did not require Institutional Review Board (IRB) approval because it was a project designed to develop educational materials and, thus, did not fall under the category of activities requiring review under the Common Rule. The interview with the cognitively normal older adults was approved by the University of Pennsylvania IRB and all participants gave informed consent to participate.

## Results

### Expert identification

A total of 21 individuals were contacted (10 informed consent experts, 11 amyloid imaging experts); 14 agreed to participate, 1 refused, and 6 did not respond. Twelve experts completed a round 1 interview (six amyloid and six informed consent); two who had agreed to participate could not be reached for interview. Individuals who did not complete interviews were not included in the remainder of the study. Ten experts completed the round 2 survey (five amyloid and five informed consent), with two experts not responding, and nine experts completed the round 3 survey (five amyloid and four informed consent).

Members of the expert panel came from a variety of disciplines and organizations in the U.S. and Europe. Experts from positions in academia, clinical practice, industry and government were included. The amyloid imaging experts included primarily clinical neurologists, as well as radiologists, psychiatrists and neuroscientists. The informed consent experts included individuals with backgrounds in philosophy, clinical psychology, law, genetic risk assessment and disclosure, and research and medical ethics. The expert panel and our investigative team included members of the Society of Nuclear Medicine and Molecular Imaging and the Alzheimer’s Association Amyloid Imaging Task Force who produced the Appropriate Use Criteria for Amyloid PET imaging [6].

### The Delphi process

Interviews in Round 1 produced 207 unique suggestions regarding the disclosure of amyloid results in the context of an AD prevention trial: 93 items related to topics to discuss, 55 to participant assessments, 39 to disclosure process methods and 20 to materials to utilize.

Responses on the Round 2 surveys indicated consensus to retain 70 items related to topics to discuss, 25 items related to participant assessments, 29 items related to disclosure process methods and 8 items related to materials to utilize. We removed items that are standard practice in clinical trials (such as obtaining informed consent) and then used the survey responses to develop the Amyloid Imaging Disclosure Process brochure and instructional manual. The manual is displayed in Table 1.

**Table 1:** **Amyloid imaging disclosure process instructional manual**

**Best practice**	**Details**
Step 0: Prior to in-person screening	
Send Amyloid Imaging Disclosure Process brochure prior to consent.	Brochure content descriptions are provided in Table 2.
Step 1A: Education and informed consent	
Assess knowledge of study and role of amyloid imaging in study.	Use this information to structure review of the brochure.
Assess motivation for joining study.	Example questions:
	Tell me what you know about an amyloid PET scan?
	Why are you interested in having an amyloid PET scan?
	Why are you interested in joining the study?
Conduct educational session.	Cover brochure contents and tailor based on participant’s prior knowledge.
	Study staff should be skilled in communication.
	Explain meaning of elevated amyloid on PET scan, clarify that this does not necessarily mean that an individual will develop symptoms of Alzheimer’s disease
Assess understanding of brochure.	Use “teach back” method: “Can you tell me in your own words what we just talked about?”
	Focus on understanding of amyloid imaging and its role in study.
Step 1B: Screening assessments	
Screen for anxiety and depression (for example: State-Trait Anxiety Inventory (STAI) [12] and Geriatric Depression Scale (GDS) [13])	Decisions on eligibility will be study-specific and involve investigator’s clinical judgment.
Step 2: Amyloid PET scan	
Participant undergoes amyloid PET scan.	Conduct imaging on a separate day from consent.
	Do not disclose results on the day of imaging.
Step 3A: Amyloid status disclosure - pre-disclosure	
Assess mood.	Investigator/study staff should be skilled in communication and recognition of distress.
(For example: STAI and GDS)	
Assess recent life stress.	
Assess willingness to receive result.	If concerns arise, discuss possibility of delaying disclosure.
Step 3B: Amyloid status disclosure	
Disclose amyloid status using language from the brochure.	Disclose in-person, with time for questions.
	Give participant option of having family member or friend present.
	Provide a written summary.
Assess understanding of amyloid status result.	Example questions:
	What does that mean to you?
	Do you have any questions about your result?
Step 4: Post-disclosure follow-up	
Conduct follow-up phone call one to three days post-disclosure.	Assess well-being, distress, and impact of disclosure. (for example: Impact of Event Scale (IES) [14])
	Answer questions.
	Create appropriate follow-up plan based on participant’s responses.
Step 5: Follow-up over study course	
Assess anxiety, depression, impact of disclosure.	Study protocol should specify frequency of assessments and plans for additional monitoring if distress is observed.
(for example: STAI, GDS, IES)	

Round 3 survey responses showed high clarity ratings for brochure sections. Mean section clarity ratings ranged from 3.67 to 4.5, with only two sections receiving mean clarity ratings lower than 4. We revised brochure sections based on clarity ratings and specific expert comments. In particular, we simplified language and added text explaining that a person could have an elevated amyloid scan and never develop AD dementia.

### Test for readability with cognitively normal older adults

A convenience sample of five cognitively normal adults (all who were approached agreed to participate) completed in-person interviews using the template brochure. All participants were non-Latino whites and 80% were women. Average age was 89 ± 3.8 years (range 82 to 91). Participants averaged 14.4 ± 2.6 years of education (range 12 to 18).Testing of the generalized version of the brochure, without specific reference to A4, showed that older adults found the brochure clear and comprehensible, and were able to summarize key points after reviewing the document. We made minor changes based on their recommendations, including removing redundant wording and adding information about the progression of AD symptoms over time. Topics and key points from a template version of the brochure which can be adapted for any secondary prevention trial are displayed in Table 2.

**Table 2:** **Amyloid imaging disclosure process brochure template**

**Brochure topic**	**Key points**
What is the (insert name of secondary prevention trial (for example: A4 study)) Trial?	● This should be study specific and include basic enrollment criteria, objective, and study design
Why is this (insert name/description of intervention) being tested?	● This should be study specific and include basic explanation of the intervention’s mechanism and safety profile
What will happen if I enroll in the (insert name of secondary prevention study) Trial?	● This should be study specific and include information about the screening and enrollment process and study procedures
What is Alzheimer’s disease?	● Alzheimer’s disease is a brain disease.
	● It is the most common cause of dementia.
	● Common symptoms of dementia caused by Alzheimer’s disease are problems with memory and thinking that impair a person’s ability to do their usual and everyday activities.
	● As persons with Alzheimer’s disease develop symptoms, the first to appear are memory and thinking problems that are bothersome but do not interfere with daily activities. Over time, usually several years, as these problems worsen, the person develops dementia.
What is amyloid?	● Amyloid is a protein in the brain
	● In Alzheimer’s disease, amyloid builds up and brain function gets worse
	● Amyloid can sometimes be detected years before a person has noticeable memory problems.
How do we know whether someone has brain amyloid?	● An amyloid PET scan measures brain amyloid.
What does having a brain amyloid scan involve?	● An injection of a radioactive drug.
	● The scan measures the level of amyloid in your brain.
What does an elevated level of brain amyloid mean?	● An ‘elevated amyloid’ result:
	- means that amyloid plaques are present in your brain.
	- does not mean you now have Alzheimer’s disease dementia or that you will ever get Alzheimer’s disease dementia.
	- means you may be eligible to join Alzheimer’s prevention trials that will test anti-amyloid therapies.
	● Recent studies suggest that elevated levels of amyloid may increase your risk of developing Alzheimer’s disease dementia in your lifetime.
Is an elevated level of amyloid like other medical risks?	● The relationship between elevated amyloid and Alzheimer’s disease dementia is similar to the relationship between high cholesterol and heart disease.
	● Many factors protect a person from developing memory or thinking problems even if they have elevated levels of amyloid.
	● Good general health and a healthy lifestyle are known to lower the risk of Alzheimer’s disease dementia.
What does a not elevated level of brain amyloid mean?	● A ‘not elevated’ amyloid result:
	- means that it is unlikely you have amyloid plaques in your brain at this time.
	● A person who has a ‘not elevated’ amyloid level could develop:
	- an ‘elevated’ level in the future.
	- Alzheimer’s disease dementia in the future.
Why is a brain amyloid scan necessary to participate in the (insert name of secondary prevention study) Trial?	● The trial will test whether an amyloid lowering drug given to people with elevated amyloid will lower the amount of brain amyloid and also prevent or slow declines in memory.
	(This text can be edited to fit the specific secondary prevention trial’s goals and intervention)
	● The brain amyloid scan will indicate whether a person has elevated amyloid and is then eligible to participate in the trial.

## Discussion

Disclosing AD biomarker results to cognitively normal older adults in a research setting raises clinical and ethical challenges and has been discouraged due to prognostic uncertainty and lack of clinical utility. However, the design of secondary prevention trials that will enroll only cognitively normal individuals with Alzheimer’s biomarkers necessitates disclosure, and so a process to safely and effectively disclose such results is urgently needed [8,15,16]. Creating a safe and effective disclosure fulfils the principles of respect for individuals’ autonomy (competent participants should make a fully informed and voluntary decision), and non-maleficence, (they should not be harmed by the information).

To fill this gap in knowledge, we utilized a modified Delphi procedure to develop expert consensus on topics to be discussed and best practices for amyloid imaging result disclosure to cognitively normal research participants. The best practices include pre- and post-imaging recommendations and are compiled in the ‘Amyloid Imaging Disclosure Process Instructional Manual’. Text covering the topics to be addressed is included in the ‘Amyloid Imaging Disclosure Process Brochure’. Versions of both documents are being utilized in the A4 Study and can be adapted for use in other prevention trials.

The disclosure process includes a detailed pre-consent educational session focused on informing participants of what is known and unknown about amyloid imaging, detailing possible imaging results and their implications, and providing context in the form of basic information about Alzheimer’s disease and its risk factors. The process also includes careful participant screening, mood monitoring, and a protocol for monitoring and addressing distress. Mood monitoring should include assessment of depression, anxiety, and the impact of the disclosure event using standard, validated instruments. Strategies to assess comprehension, such as the ‘teach-back’ method, are addressed, as well as required research staff skills, including clear communication and ability to recognize and respond to participant distress.

This amyloid imaging disclosure process was designed with the goal of minimizing the risks of disclosure. These risks, which have been described elsewhere [17-20], include potential psychological reactions such as anxiety and depression, misunderstanding of the result and its implications – in particular, believing that an elevated amyloid scan indicates a clinical diagnosis of Alzheimer’s, stigma in interpersonal relationships, discrimination in realms such as insurance and employment, and effects on cognitive performance due to stereotype threat.

The process we developed is similar in many respects to a proposed process for disclosing PET amyloid imaging to persons with mild cognitive impairment [21-23]. Those investigators developed disclosure materials through consultation with a panel of experts, piloted the materials in mock disclosures to MCI patients and their family members, and conducted follow-up focus groups with MCI patients and family members for additional feedback. They found that patients and family members generally understood the imaging results and were satisfied with the disclosure process. Specific recommendations included conducting pre-test counseling, using clear graphics, reviewing patient’s scan images during the disclosure session, providing take-home materials with follow-up information, conducting phone follow-up after disclosure, and communicating with primary care providers to facilitate treatment planning [21-23]. The A4 study does not show participants’ scan images or provide specific quantitative results from the amyloid imaging. Reviewing the PET scans during disclosure was suggested in Round One interviews, but we did not find consensus to do so in later Delphi rounds. Further research is needed to determine participants’ desire to view their images, how to present visual or quantitative information from the scans, and what the impact of this additional information is.

The development of this disclosure process and documents is only a first step. Research is needed to analyze the actual safety and effectiveness of this process, including how well it works (for participants and trial staff), and what are the effects (psychological, social, legal, cognitive, behavioral) of amyloid imaging result disclosure on cognitively normal research participants who have and do not have evidence of elevated amyloid accumulation. The Risk Evaluation and Education for Alzheimer’s Disease (REVEAL) studies have shown that information about genetic risk for Alzheimer’s can be safely and effectively disclosed to research participants with minimal harm [24-27]. The process developed here is quite similar to REVEAL Study methods, although key differences between genetic risk and biomarker positivity may lead to differences in participant reactions. Data on the impact of disclosure will be collected through a variety of measures – many adapted from the REVEAL Study – within the A4 study, as well as through an add-on study involving qualitative interviews with A4 participants and individuals who screen out of A4 due to not having elevated amyloid. These interviews will address the impact of receiving amyloid imaging results, including behaviors adopted since learning amyloid imaging results, sense of self, experiences of sharing the results with others, and experiences of discrimination or stigma. The data from A4 and the qualitative interviews will be analyzed to validate and refine the disclosure process.

Our study is limited by drop out or non-participation among five experts over the course of the study, who, it is possible, had views markedly different from the experts who did respond and so would have shaped our results in a different manner. Second, while we did test the materials in a group of cognitively normal older adults, our sample was relatively well-educated and not ethnically diverse. Evidence to inform whether the materials achieve the goal of safely disclosing results to older adults awaits the actual implementation in the A4 Study, a process that is ongoing and results are expected by the close of 2015. Further study is necessary to determine whether learning this information under circumstances outside of a clinical trial testing a potentially beneficial intervention would be safe. Those results, as well as additional data on the clinical significance of amyloid imaging in cognitively normal older adults, will likely require further revisions of this process.

## Conclusions

We utilized a modified Delphi Method to develop a document for use with potential AD secondary prevention study participants to describe the process of amyloid imaging and the implications of amyloid imaging results; and a disclosure process with attention to ongoing monitoring of both mood and safety to receive this information. This document and process will be used in the A4 Study and can be adapted for other research settings. Evidence to inform whether the materials achieve the goal of safely disclosing results to older adults awaits actual implementation of the process in A4 and other studies.

## Abbreviations

A4: anti-amyloid treatment in asymptomatic Alzheimer’s
AD: Alzheimer’s disease
ADCS: Alzheimer’s disease cooperative study
ADNI: Alzheimer’s disease neuroimaging initiative
FDA: Food and Drug Administration
IRB: institutional review board
MCI: mild cognitive impairment
NAPA: National Alzheimer’s project act
PET: positron emission tomography
REVEAL: Risk evaluation and education for Alzheimer’s disease
